# Acoustic compression in Zoom audio does not compromise voice recognition performance

**DOI:** 10.1038/s41598-023-45971-x

**Published:** 2023-10-31

**Authors:** Valeriia Perepelytsia, Volker Dellwo

**Affiliations:** Department of Computational Linguistics, University of Zurich, Andreasstrasse 15, 8050 Zurich, Switzerland

**Keywords:** Psychology, Human behaviour

## Abstract

Human voice recognition over telephone channels typically yields lower accuracy when compared to audio recorded in a studio environment with higher quality. Here, we investigated the extent to which audio in video conferencing, subject to various lossy compression mechanisms, affects human voice recognition performance. Voice recognition performance was tested in an old–new recognition task under three audio conditions (telephone, Zoom, studio) across all matched (familiarization and test with same audio condition) and mismatched combinations (familiarization and test with different audio conditions). Participants were familiarized with female voices presented in either studio-quality (N = 22), Zoom-quality (N = 21), or telephone-quality (N = 20) stimuli. Subsequently, all listeners performed an identical voice recognition test containing a balanced stimulus set from all three conditions. Results revealed that voice recognition performance (dʹ) in Zoom audio was not significantly different to studio audio but both in Zoom and studio audio listeners performed significantly better compared to telephone audio. This suggests that signal processing of the speech codec used by Zoom provides equally relevant information in terms of voice recognition compared to studio audio. Interestingly, listeners familiarized with voices via Zoom audio showed a trend towards a better recognition performance in the test (p = 0.056) compared to listeners familiarized with studio audio. We discuss future directions according to which a possible advantage of Zoom audio for voice recognition might be related to some of the speech coding mechanisms used by Zoom.

## Introduction

Voice recognition is essential for human communication since it allows us to structure and understand the linguistic content in speech^1–3^, facilitates speech recognition in noisy environments^4,5^, and when impaired, hinders successful social interactions^6^. Until recently, familiarization with voices and thus learning of speaker-specific voice features usually occurred via face-to-face communication or voice-to-voice over the telephone, which is why both these conditions received significant attention in voice recognition studies^7–11^. However, much of personal, business, and education communication now occurs online in a digital format. Therefore, we often are exposed to and learn new voices not directly via face-to-face communication, but from the digital communication modes, as well as digitized and compressed audio signals. Digital audio plays a significant role in mobile communications, internet telephony (i.e., Voice Over Internet Protocol, VoIP), voicemail, videoconferencing, as well as gaming and audio streaming over the Internet. All of these applications use compression algorithms (i.e., codecs) to efficiently compress and send audio and video data over the Internet. However, it is unclear how different audio codecs affect human voice recognition, in particular those used in videoconferencing applications, among which Zoom (Zoom Video Communications, Inc.) is the leading one. Considering that crucial data for voice recognition, such as those used in forensic investigations of voice identity, now frequently come from Internet audio compression conditions, it is crucial to understand how the recognition performance of internet audio compares to that of studio and telephone audio.

### Human voice recognition via telephone audio

Most studies on voice recognition under telephone audio conditions focused on the effects of traditional landline telephones; much less work was done on mobile telephony. The dominant limitation of telephone audio (both landline and mobile) is its reduced bandwidth compared to studio audio^7,12^. Landline telephones limit the audio bandwidth to approximately 300–3400 Hz (variation between individual systems exist); henceforth narrow-band audio. In addition to bandwidth limitations, telephone transmission also distorts the remaining frequencies in the reduced bandwidth in various ways: for example, frequencies closer to the lower cutoff are shifted upwards, while frequencies closer to the upper cutoff are shifted downwards^7,12–14^. Modern mobile and especially Internet telephony use broader bandwidth of approximately 50–7000 Hz, henceforth wide-band audio, which might be more beneficial for voice recognition compared to narrow-band. However, mobile phone transmission affects acoustic features more drastically than landline telephony, since transmission characteristics may dynamically change during mobile calls due to changing network conditions^13–15^. Telephone audio quality may also vary depending on the technical characteristics of the devices used during calls^16^. Furthermore, telephone transmission is subject to various environmental effects, such as background noise (e.g., traffic)^13^ and voice production effects, such as the so-called ‘telephone voice’, a speaking register characterized by increased loudness and higher F0, which speakers adopt when speaking on the phone^17^.

Most studies investigating the effects of telephone transmission on voice recognition by human listeners showed that listeners perform worse in telephone compared to studio audio or direct presentation^8,9,18,19^, and that voice recognition via telephone audio is challenging even for members of close social networks^20^. Several experiments, however, did not find differences between recognition performance via telephone and studio audio^10,21,22^, likely because of differences in telephony characteristics (landline vs. mobile telephony), retention intervals between familiarization and test, as well as stimuli and task types. One study exploring the effect of telephone and direct presentation on voice identification accuracy found that voice recognition performance was slightly better via telephone than via direct presentation^23^. However, the overall recognition rates in this experiment were poor across both telephone and directly presented condition. Interestingly, voices sound more similar to the listeners in telephone audio potentially since much of speaker-distinguishing spectral information is lost due to transmission limitations discussed above^11,24^. While most studies investigated the effect of telephone audio in the *test*, several studies investigated how *familiarization* with voices via telephone audio affects subsequent recognition compared to familiarization with studio audio. Results are inconsistent, with some studies showing no effect of telephone in familiarization^10,21^, and some showing a detrimental effect of telephone familiarization on subsequent recognition^8^, possibly due to varying experimental designs.

Despite much of research on telephone and studio audio, very little is known about how human voice recognition is affected in videoconferencing audio, which has different transmission characteristics compared to telephone audio in terms of bandwidth and speech codec processing (see the following section). Video conferencing applications such as Zoom, Microsoft Skype, Microsoft Teams, and others gained much popularity during COVID-19 pandemic, and they continue to be widely used for different types of communication today. One prominent feature of videoconferencing is its multimodality, i.e., the availability of both video and audio signals. It has been shown that voice recognition can be affected by visual cues in different ways. After short familiarization with voices in the presence of corresponding faces, the so called ‘face overshadowing effect’ may occur, a phenomenon by which the presence of the face leads to poorer recognition results in audio only recognition compared to conditions when only the voice is present during familiarization. However, the relationship between audio and visual cues on voice recognition is complex^25–28^, and several studies revealed that with longer familiarization durations there are actually strong advantages of face presence in learning voices^29^. Therefore, to ensure the comparability of audio conditions, we focus only on audio cues in this study and investigate human voice recognition via videoconferencing audio (namely, Zoom audio) in comparison to studio audio and telephone audio.

### Voice processing information has not yet been part of audio codec assessment

During calls, videoconferencing applications transmit audio (and video) data over the Internet. Before transmission, analog speech signal must be efficiently encoded (i.e., digitized and compressed) to reduce the amount of transmitted information with minimal quality loss. This is achieved by means of audio codecs. After encoding, the data is converted into a stream of discrete packets and transmitted to the destination, where it is decompressed and converted back into the analog signal. Because network conditions may change dynamically during a call, some data packets may arrive late or even get lost, which might result in changes in voice quality, as well as distorted or unintelligible speech. Codec is the thus main factor that directly affects the quality of the transmitted signal, since it determines the acoustic bandwidth and the bitrate of the transmitted signal, as well as dynamically changes these parameters depending on the network conditions^30^.

Zoom digitizes the speech signal by means of the open source Opus codec, which incorporates technology from Skype’s SILK codec and Xiph.Org’s CELT codec^31^. Opus was developed for interactive speech and music transmission over the Internet, and it is a multifunctional and highly adaptive codec supporting different kinds of audio from narrow-band mono speech to full-band (20–20,000 Hz) stereo music at a wide range of bitrates^31^. To achieve good compression, Opus uses both Linear Prediction (LP) layer, which is based on SILK codec, and Modified Discrete Cosine Transform (MDCT) layer, which is based on CELT codec^31^. The main purpose of LP-based layer in SILK is to reduce the bitrate by reducing the residual energy, which is achieved by the Burg’s method, since it provides higher prediction gain compared to other techniques^31^. The main principle behind the CELT-based MDCT layer is that the MDCT spectrum is split into bands following the Bark scale, i.e., a psychoacoustic scale which closely follows the frequency resolution of the human ear^31^. Opus uses LP layer to encode speech, since LP techniques are more efficient for coding lower frequencies than transform domain techniques, whereas MDCT technique is used to code higher speech frequencies or music (i.e., for bandwidth 8 kHz or higher)^31^. At any given time, either one (SILK-based LP or CELT-based MDCT) or both layers may be active to adapt to changing network conditions during transmission^31^. Opus employs SILK mode for low bitrate transmission with narrow-band (i.e., 4 kHz), medium-band (i.e., 6 kHz), and wideband (i.e., 8 kHz) audio bandwidth; it employs a hybrid mode (SILK + CELT) for super wideband (12 kHz bandwidth) or full-band (20 kHz bandwidth) speech at medium bitrates; lastly, CELT-only mode is employed for low delay speech transmission and music transmission^31^.

Due to availability of both SILK and CELT layers, Opus can operate at a wider range and offer comparable or even better performance than several other state-of-the-art voice codecs^32^. Crucially, the quality assessment of Opus both with human listeners and computer algorithms focused on assessing overall audio and speech quality and intelligibility, but not voice recognition performance^33,34^. Therefore, the effect of Opus processing on human voice recognition performance remains unclear.

Assessing perceptual quality of compressed audio is important for developing modern multimedia systems such as audio streaming over the Internet, gaming, mobile telephony, VoIP technology, and others. Standardized methods for evaluating the audio codecs performance and the resulting quality of coded audio typically target overall audio quality and speech intelligibility (for reviews, see^35,36^). The International Telecommunication Union (ITU) recommends using a five-point rating scale for collecting listener judgements of perceived audio quality (for example, see the most widely used ITU-R BS.1284-1, ITU-R BS.1116, ITU-R BS.1534-1, and ITU-T P.800 recommendations for evaluating perceptual audio quality by human listeners). In the standardized listening experiments, listeners rate the audio quality coded by different codecs on the scale from 1 (‘bad’) to 5 (‘excellent’) or audio impairment on the scale from 1 (‘very annoying’) to 5 (‘imperceptible’), depending on the experimental design. Mean opinion scores (MOS) are derived from these ratings and reflect overall audio quality or the severity of introduced audio impairments. Listening tests are time consuming and costly to implement, and as a result, assessing quality of compressed audio is mostly done using computer-based algorithms.

Therefore, the impact of different speech and audio codecs on voice recognition in human listeners received very little attention. Gallardo et al.^37^ studied human voice recognition in speech transmitted through several narrow-band and wide-band codecs to explore the benefit of extended bandwidth on voice recognition in human listeners. They found that wide-band speech signal (from G.722 and adaptive multi-rate wideband codecs) was beneficial for human voice recognition compared to narrow-band signal (from G.711, adaptive multi-rate narrowband, and global system for mobile communications enhanced full rate codecs). A subsequent study^16^ compared the effect of different codecs on human voice recognition in narrow-band, wide-band, and super-wide band coded speech. Using both male and female speakers, they found that wide-band signal is better that narrow-band signal, but super wide-band offers no improvement for human voice recognition compared to wide-band signal. This means that signal information below 7 kHz contains sufficient information for successful voice recognition.

On the other hand, speaker recognition performance was tested for various codecs using automatic speaker recognition (ASR) systems^30,38–41^. Generally, it was found that higher bitrates^38,42^ and extended bandwidth^41,42^ improve performance of automatic systems. However, it is unclear whether further improvements in signal quality due to even further bandwidth extension and higher bitrates result in the significantly better performance of ASR systems as speech information becomes sparser with increasing frequency higher than about 7 kHz. For example, it was shown that bandwidth extension from wide-band to super-wide band leads to a significant improvement in performance of ASR systems compared to wide-band speech, but only in female speakers^41^. Finally, codec mismatch between training and test typically degraded voice recognition performance compared to codec matched condition in ASR systems^38–40^.

Understanding how well different codecs transmit speaker-specific information and thus how successfully human listeners can recognize voices via audio coded by different codecs is relevant both for forensic context and for general understanding of human voice recognition. Currently, many forensic cases include speech samples not from traditional landline telephony, but rather audio from the mobile and VoIP telephony, voicemail from instant messaging apps, as well as audio (and video) from videoconferencing apps, all of which use modern codecs for speech compression. Human listeners are constantly familiarized with voices over video conferencing apps thus ear-witnesses of crime are liable to give evidence on voices in the courtroom that they heard over the internet. Therefore, it is important to investigate how codec processing with varying parameters such as different bandwidths and bitrates influences voice recognition in human listeners. This is also relevant for the general understanding of the voice recognition performance to gain deeper insights into which acoustic features of the signal predominantly carry speaker-specific information. In the construction and interpretation of future voice recognition experiments using audio transmitted via the Internet, the present study will offer crucial insights.

### The present study

Here, for the first time, we carried out a comprehensive comparison of human voice recognition performance across three different audio conditions: high-quality studio audio, telephone audio, and Zoom audio. The study followed two aims: to assess the effect of audio condition in (1) familiarization and (2) in test. To address this, we conducted an online listening experiment with three groups of listeners, whereby in a between-group design, each group was familiarized with voices via either studio-quality, Zoom-quality, or telephone-quality audio. After familiarization, all listeners performed a voice recognition test (old–new judgement task), in which stimuli of all three audio qualities (studio, Zoom, and telephone) were included. Thus, the experiment included both matched (for example, studio audio in both familiarization and test) and mismatched conditions (for example, studio audio in familiarization and Zoom or telephone speech in test or vice versa).

We expect the general effect of familiarization audio, such that listeners familiarized with voices via studio audio will perform overall better in the subsequent voice recognition test in all audio conditions (Zoom, studio, telephone) compared to Zoom and telephone familiarized listeners, because studio audio contains more speaker-specific voice information compared to Zoom and telephone audio due to extended bandwidth and absence of speech codec processing. As for the effect of test audio, we expect the best performance in studio audio in test, since it provides listeners with the most of speaker-specific voice information. As for the Zoom audio in test, it is plausible that bandwidth reduction does not impair human voice recognition^16,43^. However, codec processing may result in various non-linear distortions during a call such as packet loss, packet delay, or jitter, which may affect voice recognition performance^41^. Also, Opus codec used by Zoom was not yet tested for human voice recognition, it is possible that recognition performance via Zoom audio in test will be worse than via studio audio but better than via telephone audio. Given a generally subjectively perceived higher quality of Zoom audio compared to landline telephone, this assumption is plausible. While Opus settings may change during a call depending on network condition, number of users, and other factors, we ensured that Zoom audio quality was homogeneous during the recording (see “Methods” section). As for the telephone audio in test, we expect the lowest recognition performance compared to studio and Zoom audio, since previous studies showed that landline telephone audio results in lower recognition performance compared to studio audio^8,9,19^.

## Results

### Sensitivity (dʹ)

**Figure 1:**
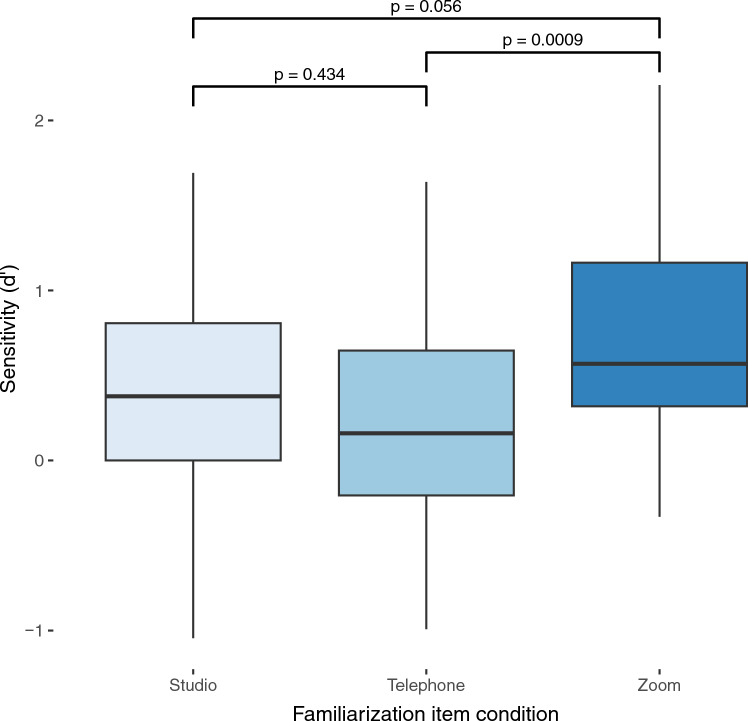
Boxplots showing median, range, and inter-quartile range of dʹ by familiarization groups. Significance levels are indicated for each pairwise comparison.

We first tested the two-way interaction between audio quality in familiarization and audio quality in test on dʹ to ensure that voice recognition performance in the test factor is not modulated by different levels of the familiarization factor. The interaction was not significant (F (4, 120) = 0.52, *p* = 0.718, η^2^ = 0.02) suggesting that listeners’ performance in different audio conditions in voice recognition test was not modulated by audio quality during familiarization. Consequently, for the subsequent analysis, we analyzed main effects of familiarization and test individually. The main effect of familiarization audio on dʹ was significant F(2, 60) = 7.46, *p* = 0.0013, η^2^ = 0.2) suggesting that listener groups differed in overall voice recognition performance, irrespective of audio quality in the test. We used pairwise t-tests for post hoc comparisons of the main effect of familiarization audio on dʹ with Bonferroni correction for multiple comparisons. The results indicate that listeners familiarized with the voices via Zoom audio performed significantly better compared to listeners familiarized with the voices over the telephone audio (*p* < 0.0009, d = 1.24) (Fig. 1). Furthermore, there was a trend of Zoom-familiarized listeners to perform better even compared to the studio-familiarized listeners (*p* = 0.056, d = 0.73). Lastly, there was no significant difference between the studio-familiarized listeners and telephone-familiarized listeners (*p* = 0.43, d = 0.44) on their performance in the test (Fig. 1).

**Figure 2:**
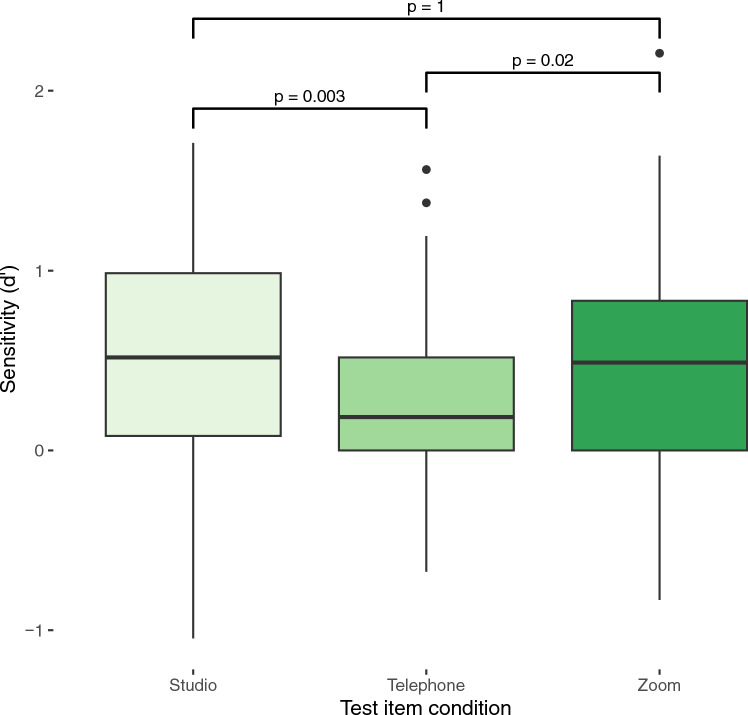
Boxplots showing median, range, and inter-quartile range of dʹ by audio condition in voice discrimination test. Significance levels are indicated for each pairwise comparison.

The main effect of audio quality on dʹ in voice recognition test was also significant (F(2, 124) = 5.62, *p* = 0.0046, η^2^ = 0.08) suggesting that listeners’ performance differed significantly depending on the audio quality in the voice recognition test irrespective of their familiarization audio. Pairwise t-tests with Bonferroni correction for multiple comparisons were used to perform post hoc comparisons of listeners’ performance across different channels in voice recognition test. The results indicate that listeners performed significantly better via Zoom audio in the test compared to the telephone audio (*p* = 0.02, d = 0.43) (Fig. 2). Listeners’ performance via studio audio during test was also significantly higher compared to telephone audio (*p* = 0.0034, d = 0.44). Lastly, no significant difference was found between listeners’ performance via Zoom audio and studio audio in the test (*p* = 1, d = 0.003) (Fig. 2).

### Response bias (*c*)

**Figure 3:**
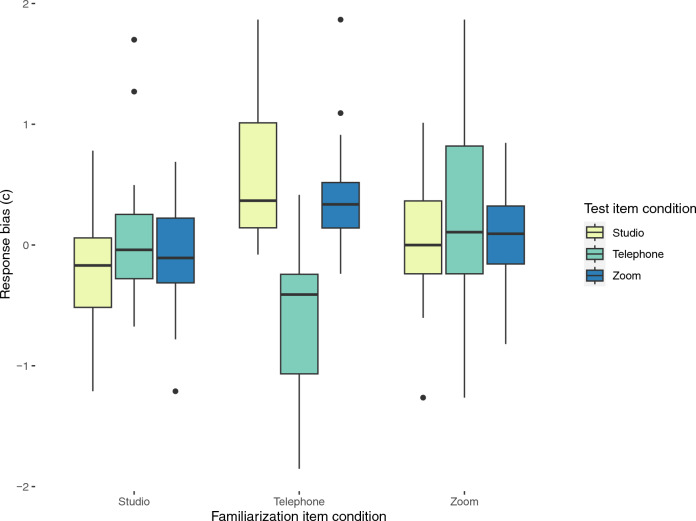
Boxplots showing median, range, and inter-quartile range of response bias (*c*) grouped by familiarization groups and audio condition in voice discrimination test.

We first tested the two-way interaction between audio quality in familiarization and audio quality in test on *c* to ensure that response bias in the test factor is not modulated by different levels of the familiarization factor. The interaction between familiarization audio and test audio on c was significant (F (4, 120) = 14.85, *p* < 0.001, η^2^ = 0.33) suggesting that listeners’ response bias across different audio qualities in the test varied depending on the audio quality during familiarization (Fig. 3). Post hoc pairwise comparisons were performed for the test audio within each familiarization group using pairwise t-tests with Bonferroni correction on *c*. No pairwise comparisons were significant within the Zoom- and (all *p*-values > 0.05, all d values < 0.2) studio-familiarized groups (all *p*-values > 0.05, d values range between 0.21 and 0.35) suggesting that listeners’ response bias in these familiarization groups did not differ depending on the audio quality they heard during voice recognition test (Fig. 3). However, listeners familiarized with voices via telephone audio were significantly more biased towards responding ‘new’ when they heard either Zoom (*p *< 0.0001, d = 1.33) or studio audio (*p* < 0.0001, d = 1.39) compared to when they heard telephone-quality stimuli. Bias for Zoom audio in the test did not differ from bias for studio audio in this group of listeners (*p* = 1, d = 0.41) (Fig. 3).

## Discussion

This study investigated voice recognition performance via studio, Zoom, and telephone audio. We studied the effect of audio quality in familiarization on subsequent voice recognition performance, as well as the effect of audio quality in test on human listeners’ voice recognition performance, quantified with dʹ and *c*. Interestingly and unexpectedly, our results revealed no difference in recognition performance between studio- and Zoom-familiarized listeners. In addition, we found a tendency for performance to be better when listeners were familiarized with the voices under Zoom compared to studio audio, however, given the diversity of listeners and equipment between the listener groups, this tendency might be a result of random variability between groups. It will be interesting to test possible advantages of familiarization over Zoom audio in more controlled experimental designs in the future. Lastly, we observed no differences between studio- and telephone-familiarization, which is mostly in line with previous work^10^. This suggests that neither too much (i.e., studio audio), nor too little speaker-specific detail about voices (i.e., telephone audio) during familiarization is beneficial for human voice recognition if familiarization period is brief. When familiarization is brief (like in our experiment), too much speaker-specific voice detail might be distracting and not helpful for forming stable voice representations. With increasing familiarization time, however, more speaker-specific detail might be beneficial for forming voice representations for subsequent recognition. This view is supported by findings from voice and face recognition about the effect of stimuli variability during familiarization on subsequent recognition showing that variability in stimulus material does not always pose challenges and—on the contrary—might be beneficial for learning new identities^44–47^.

In the test, recognition via both Zoom and studio audio was significantly better compared to telephone audio. Our results therefore align with previous research showing that signal bandwidth plays a critical role in voice recognition, since speaker-specific voice properties are distributed across frequency domain^48^. They also suggest that extending the bandwidth from wide-band (50–7000 Hz) to super wide-band (50–14,000 Hz) or unprocessed full-band speech offers no voice recognition advantage for human listeners, in line with previous research^16,43^. It is evidently the case that Opus encoding algorithms contributed to the improved recognition performance over Zoom audio compared to telephone audio, since Opus uses both LP-based layer based on source-filter model to efficiently encode lower speech frequencies (i.e., up to 8 kHz bandwidth) and MDCT-based layer to encode higher speech frequencies (i.e., higher than 8 kHz bandwidth)^31^.

Overall, our findings indicate that despite acoustic compression introduced to Zoom audio by bandwidth limitations and codec processing, voice recognition over Zoom audio is no better or worse than over studio audio, especially when exposure to voices is brief as in the present experiment. Zoom audio undergoes a wide spectrum of signal processing mechanisms (see “Introduction” section) by which the speech signal is compressed, and information is irreversibly removed. Crucially, however, our results suggest that this compression of the signal does not compromise voice recognition performance. On the contrary, the group that was familiarized with Zoom audio even showed the tendency for a better recognition performance. As stated above, it is thus possible—and this remains to be tested in future research—that the speech processing mechanisms involved in Zoom are potentially beneficial for learning voices. What may be the reasons for that? Some of the most dominant dimensions responsible for successful voice recognition are characteristics about the source (the signal produced in the larynx) and the filter (the source signal changes as an effect of resonances in the vocal tract). Acoustically, source signal details manifest in fundamental frequency characteristics, and filter detail—in spectral envelope. Algorithms like Linear Predictive Coding involved in the Zoom audio codec (see “Introduction” section), split the audio signal into source and filter information for more efficient signal transmission purposes. Both source and filter are thus coded as abstract representations of the original natural features they were derived from. It is possible that precisely this mechanism of turning the information of source and filter—which is crucial for voice identity—into abstract representations also leads to audio representations of these signals that better contain the abstract speaker characteristics of these dimensions. For this reason, compression mechanisms involving source-filter separation and individual coding of this information may reduce irrelevant acoustic variability, leading to a more precise prototype representation of speakers’ voices that supports the acquisition of these voices. That would also explain why this information plays more of a role in voice learning because learning from more prototypical information, in particular when the familiarization period is brief, helps in recognition, however, when this information has not been previously acquired it does not necessarily enhance recognition.

In terms of listeners’ response bias, we found no differences across test audio conditions in Zoom- and studio-familiarized listeners, meaning that familiarization with Zoom or studio audio did not moderate listeners’ response bias in different audio conditions in test. Interestingly, we observed differences in response bias across test audio conditions only in telephone-familiarized listeners. When this group heard telephone-quality stimuli in the test, they were significantly biased towards responding the voice is ‘old’ (i.e., familiar). This supports previous findings that voices sound more similar to the listeners via telephone audio compared to studio audio^11^, potentially because much of speaker-distinguishing information (such as higher formant frequencies above F3 or F4 and much of fricative energy) is lost due to bandwidth limitations and telephone codec processing^11^. Furthermore, when telephone-familiarized listeners heard either studio or Zoom stimuli in the test, they were significantly more biased towards responding that the voice is unfamiliar to them. This is also in line with previous results showing that voices sound more different to listeners when they hear stimuli pairs of mixed audio qualities (i.e., telephone and full bandwidth) compared to pairs of same audio qualities (i.e., only telephone or only full-bandwidth)^11^. Voice differences and audio quality differences are likely conflated for listeners, which is why they cannot solve the voice recognition task, because they are unable to separate differences in voice characteristics from differences in transmission characteristics^11^. Lastly, we found no difference between bias towards studio and towards Zoom audio in test for telephone-familiarized listeners suggesting that differences in audio quality between telephone and studio on one hand and telephone and Zoom on the other hand were perceived as equally differing from telephone audio quality.

To conclude, we found that learning voices via Zoom audio leads to better voice recognition than learning them via telephone audio and even potentially via studio audio. We also found that in test, listeners recognize voices equally well via Zoom and studio audio. This indicates that acoustic compression of Zoom audio such as bandwidth limitation and codec processing do not degrade listeners’ performance in test compared to studio audio. Future work will show whether the signal processing mechanisms involved in Zoom can enhance voice individuality information to enhance the voice acquisition and recognition process. For methodological reasons we have only tested female voices in this research. It would further be interesting to test in the future whether speaker sex impacts the voice recognition performance in particular under the type of coding used in Zoom transmission because codecs can have variable influences on male and female voices^49, 50^.

## Methods

### Speakers

We recorded nine female native speakers of Zurich German, i.e., the Alemannic dialect spoken in the city and in most parts of the Canton of Zurich. All speakers were between 22 and 27 years old (mean age = 24.6 years), born and raised in the Canton of Zurich and spoke Zurich German daily at home. None of them reported speech, language, or hearing impairments. All speakers gave written informed consent prior to the recording and received monetary compensation for their participation.

### Listeners

In total, 63 native speakers of Swiss German (18 male) participated in the study: 22 listeners in studio familiarization group, 21 listeners in Zoom familiarization group, and 20 listeners in telephone familiarization group. All were between 18 and 35 years old, born and raised in Switzerland, and none of them reported speech, language, or hearing impairments. The listeners were recruited from the student population of the University of Zurich via the University of Zurich student portal and e-mailing lists. All listeners gave an informed consent before participating and received monetary compensation for their participation. The study was approved by Ethics Committee of the Faculty of Arts and Social Sciences at the University of Zurich. The research was performed in accordance with the Declaration of Helsinki.

### Materials

Studio-quality stimuli were produced as follows. Nine female speakers described above were recorded reading 75 sentences^51^, which comprised in total 9 speakers × 75 sentences = 675 sentences. There were 25 sentences with subject–verb–object structure, 25—with subject relative clauses, and 25—with object relative clauses. The sentences were semantically unpredictable, with the average duration of 2.9 s (standard deviation 0.33 s). The speakers read the sentences in Swiss Standard German^52^. The recordings were done in a noise treated recording booth and saved directly onto a Macintosh computer connected with USB Pre 2 Portable Studio-Resolution Audio Interface and NT2-A Rode condenser microphone. The recordings were done in .wav format in Praat version 6.1.16^53^ (mono channel, sampling rate 44.1 kHz, 16 kbit/s bitrate).

The Zoom-quality stimuli were produced as follows. To avoid between-session variability, which would result from recording our speakers again over Zoom and then telephone, we connected two laptops via a Zoom call. We then played the studio-quality stimuli (675 sentences described above) directly from the sound card of one laptop and recorded them locally on the receiving laptop. 675 stimuli recorded this way comprised the Zoom-quality stimuli. The resulting audio stimuli had bandwidth of 12 kHz (super wide-band speech) meaning that the Opus codec was functioning in a hybrid mode. That is, both LP-based SILK and MDCT-based CELT encoders were used to encode speech, since SILK encoder is only used to code speech signals up to 8 kHz bandwidth, while CELT encoder encodes speech higher than 8 kHz bandwidth. Therefore, the output bitstream of the Opus encoding includes bits from both SILK and CELT encoders. However, these are not separable due to the use of a range coder^31^. Zoom-quality stimuli were produced in Zoom version 5.6.1.

The telephone-quality stimuli were obtained by playing the 675 studio-quality stimuli on one mobile phone and recording them on the receiving mobile phone during a phone call. Because VoIP technology is currently dominating in mobile telephony calls in Switzerland, the telephone stimuli recorded this way have broader bandwidth (8 kHz), unlike narrow-band speech recorded via landline telephony (300–3400 Hz). Therefore, we additionally band-pass filtered telephone-quality stimuli to 290–3510 Hz with the slope of 20 Hz. 675 sentences obtained this way comprised the telephone-quality stimuli. All stimuli (i.e., 3 audio conditions (i.e., studio, Zoom, and telephone) × 675 sentences = 2025 in total) were normalized to 70 dB SPL. The overview of the audio conditions is presented in Table 1.

**Table 1:** Main characteristics of audio conditions used in the current study.

Audio condition	Codec processing	Bandwidth
Studio	No	22.05 kHz
Zoom	Yes	12 kHz
Telephone	Yes	290–3510 Hz

### Procedure

Our experiment followed the design of the first two phases of the Glasgow voice memory test^54^ and consisted of two parts: familiarization and voice recognition test (old–new judgement). Table 2 presents an overview of experimental design.

**Table 2:** Design of listening experiment. During familiarization, listeners were familiarized with four female voices in either studio-, or Zoom-, or telephone-quality audio. In subsequent voice recognition test, they heard audio stimuli in all three qualities (i.e., studio, Zoom, and telephone) irrespective of the familiarization group.

Familiarization audio 4 voices × 5 sentences = 20 stimuli	Test audio 8 voices × 3 channels × 4 sentences = 96 stimuli
Group 1: Studio	All groups: Studio + Zoom + Telephone
Group 2: Zoom	
Group 3: Telephone	

When entering the experiment, listeners were randomly assigned into one of the three familiarization groups depending on the audio quality for familiarization. Group 1 was familiarized with voices via studio audio, Group 2—via Zoom audio, and Group 3—via telephone audio. First, participants did a demo section, in which they were familiarized with the procedure and task of the experiment. Female speaker heard during this demo section was excluded from the pool of speakers for subsequent familiarization and test. After this, the real experiment began. In the familiarization part, listeners were familiarized with four female voices and heard five sentence recordings per voice, which comprised 20 sentences for familiarization in total (4 speakers × 5 sentences). They pressed a button on the screen to play each stimulus, which could be heard only once. Right after the familiarization part, the voice recognition test began. In each trial, listeners heard a sentence and were instructed to indicate by pressing a button whether the voice they heard is ‘old’ (i.e., known from familiarization) or ‘new’ (i.e., not known from familiarization). For all listeners irrespectively of their familiarization audio, the test consisted of 96 trials and included stimuli from eight speakers (four known from the familiarization and four new, not known from familiarization) in all three audio conditions (i.e., studio, Zoom, and telephone). For each listener, speakers were randomly assigned into familiar, unfamiliar, and demo, such that no fixed set of speakers served only as familiar or unfamiliar for all listeners. Furthermore, each listener received a random subset of 96 stimuli drawn from a common pool of 2025 stimuli (see “Materials” section). Equal proportion of trials included studio, Zoom, and telephone audio, and the order of presentation was randomized. To avoid memory effects, stimuli with which listeners were familiarized with voices were excluded from the test. The experiment took on average 12 minutes to complete and was conducted online on Gorilla Experiment Builder platform^55^. Listeners received a link to access the experiment by email and performed the experiment at home.

### Statistical analyses

We analyzed the data using signal detection theory (SDT), which provides a general framework to systematically characterize decision-making in the presence of uncertainty^56–59^. According to SDT, participants’ performance in many perceptual tasks may be fully described by two measures: sensitivity to the signal (quantified with dʹ) and response bias (quantified with several different indices), which can independently affect participants’ performance^59^. We used dʹ and response bias measure *c* (i.e., criterion location) to analyze the listeners’ performance in our experiment.

In an old–new judgement task used in our experiment, there were four possible combinations of trial type and response (Table 3).

**Table 3:** Table of scores in old–new voice recognition task used in the current study.

Trial	Response	
	Old	New
Old	Hit	Miss
New	False alarm (FA)	Correct rejection (CR)

The experiment consisted of *signal trials,* which contained a signal (i.e., a familiar voice known from familiarization, henceforth referred to as ‘old’), and *noise trials* which presented a noisy stimulus (i.e., a novel voice not known from familiarization, henceforth referred to as ‘new’). On signal trials, responding with ‘old’ was correct and was termed *hit*, while responding with ‘new’ on this type of trial was incorrect and termed *miss*. On noise trials, responding with ‘old’ was incorrect and was termed *false alarm* (FA), whilst responding with ‘new’ was correct and labeled *correct rejection* (CR). The hit rate H is the proportion of signal trials to which participant responded ‘old’: H = P(response ‘old’|signal trials), while the FA rate F is the proportion of noise trials to which participant responded ‘old’: F = P(response ‘old’|noise trials).

Participants’ sensitivity to the signal, in this case—being able to correctly identify a familiar voice—was quantified with dʹ and calculated as the difference between the *z*-transform of hit rate and that of the FA rate (Eq. 1):

1 $${\mathrm{d}}^{\mathrm{^{\prime}}}=\mathrm{z}\left(\mathrm{H}\right)-\mathrm{z}\left(\mathrm{F}\right).$$

A dʹ value of 0 indicates an inability of the participant to distinguish signals from noise, whereas larger values indicate greater ability to separate signals from noise, with maximum possible value being + ∞, which indicates a perfect performance. The minimum possible value of dʹ is − ∞.

In the current task, participants’ response bias was conceptualized as the tendency to favor one response over the other, i.e., either ‘old’ or ‘new’. Response bias was quantified with the criterion location *c*, since *c* is unaffected by changes in dʹ, whereas other indices of response bias such as *β* are not^60,61^. Criterion location reflects the distance between the selection criterion (i.e., the participant’s threshold for giving a certain response) and the *neutral point*, where neither response is favored^59^. Since *c* is based directly on the selection criterion, and not on the likelihood ratio like *β*, some researchers recommend it instead of other indices of bias^61^. *c* is calculated as the negative value of half the sum of z(H) and z(F) (Eq. 2):

2 $$c= -0.5\left(\mathrm{z}\left(\mathrm{H}\right)+\mathrm{z}\left(\mathrm{F}\right)\right).$$

If the criterion location is at the neutral point, *c* = 0, which indicates a lack of bias. Negative values of *c* indicate a bias towards responding that the signal is present (i.e., favoring response ‘old’ in the current task), while positive values of *c* signify a bias towards responding that the signal is absent (i.e., favoring response ‘new’ in the current task)^59^.

dʹ and *c* values were calculated for each audio condition in test for each participant, therefore, each participant had 3 dʹ and 3 *c* values corresponding to three audio conditions during test. Before calculating dʹ, we corrected the values of hit and FA rates of 0 and 1 to avoid *z* scores of + ∞ and − ∞: hit and FA rates of 0 were replaced with $1/2N$, and hit rates and FA rates of 1 were replaced with $1-(1/2N)$, where N is the maximum possible number of FAs^59^. We did not exclude any participants based on their performance, that is, even if participants’ dʹ score was at 0 or below, they were included in the final sample. We adopted this strategy since it was not clear how telephone-familiarised listeners will perform in the test given that few previous studies explored the influence of low-quality familiarization on voice recognition. Thus, the final data set for analyses contained 189 dʹ values (63 listeners × 3 dʹ values) and 189 *c* values (63 listeners × 3 *c* values). To assess the effects of familiarization and test audio on voice recognition performance, we fitted a two-way mixed ANOVA with one between-subject factor (i.e., familiarization audio with three levels: studio, Zoom, and telephone) and one within-subject factor (i.e., test audio with three levels: studio, Zoom, and telephone) on *d*ʹ. We repeated the same procedure for *c*. All statistical analyses were conducted in R version 4.0.3^62^.

## Data Availability

Due to the data protection guidelines of the University of Zurich, the raw audio recordings produced during this study are not publicly available. However, mel-frequency cepstral coefficients (MFCCs) of the audio recordings are available for scientific purposes from the first author upon request. Behavioral data and analyses code are publicly available in the study’s Open Science Framework repository: https://osf.io/xrs9z/?view_only=3a4baffb23214dde94fa39ee251e950e.
